# The role of telehealth during COVID-19 outbreak: a systematic review based on current evidence

**DOI:** 10.1186/s12889-020-09301-4

**Published:** 2020-08-01

**Authors:** Elham Monaghesh, Alireza Hajizadeh

**Affiliations:** 1 Department of Health Information Technology, Student Research Committee, School of Management and Medical Informatics, Tabriz University of Medical Sciences, Tabriz, Iran; 2 Tabriz Health Services Management Research Center, Iranian Center of Excellence in Health Management, Student Research Committee, School of Management and Medical Informatics, Tabriz University of Medical Sciences, Tabriz, Iran

**Keywords:** Telehealth, Telemedicine, Coronavirus, COVID-19, Outbreaks

## Abstract

**Background:**

The outbreak of coronavirus disease-19 (COVID-19) is a public health emergency of international concern. Telehealth is an effective option to fight the outbreak of COVID-19. The aim of this systematic review was to identify the role of telehealth services in preventing, diagnosing, treating, and controlling diseases during COVID-19 outbreak.

**Methods:**

This systematic review was conducted through searching five databases including PubMed, Scopus, Embase, Web of Science, and Science Direct. Inclusion criteria included studies clearly defining any use of telehealth services in all aspects of health care during COVID-19 outbreak, published from December 31, 2019, written in English language and published in peer reviewed journals. Two reviewers independently assessed search results, extracted data, and assessed the quality of the included studies. Quality assessment was based on the Critical Appraisal Skills Program (CASP) checklist. Narrative synthesis was undertaken to summarize and report the findings.

**Results:**

Eight studies met the inclusion out of the 142 search results. Currently, healthcare providers and patients who are self-isolating, telehealth is certainly appropriate in minimizing the risk of COVID-19 transmission. This solution has the potential to prevent any sort of direct physical contact, provide continuous care to the community, and finally reduce morbidity and mortality in COVID-19 outbreak.

**Conclusions:**

The use of telehealth improves the provision of health services. Therefore, telehealth should be an important tool in caring services while keeping patients and health providers safe during COVID-19 outbreak.

## Background

Coronaviruses, a genus of the coronaviridae family, may cause illness in animals or humans [1, 2]. In humans, several coronaviruses are known to cause infections of respiratory ranging from the common cold to more serious diseases. The most recently discovered coronavirus causes coronavirus disease-19 (COVID-19) [1]. The disease originated in Wuhan, China and has kept spreading widely to other regions of the world [3]. Primitive symptoms of COVID-19 contain fever, dry cough, breathing difficulty, and boredom [4, 5]. Elderly people and those with underlying medical problems such as hypertension, heart problems, and diabetes are more susceptible to develop the disease in its form of most intensive [1]. This universal event has been announced a pandemic by the World Health Organization (WHO) [6]. A significant factor in slowing down the transmission of the virus is the “social gap” or social distancing that is made possible by the reduction of person-to-person contact [7, 8].

To reduce transmission, travel restrictions have been appointed and enforced around the world, and most cities have been quarantined [9]. However, people who are not infected with the COVID-19, especially those who are at greater risk of developing the disease (e.g. Elderly people and those with underlying diseases), should receive daily care without the risk of exposure to other patients in the hospital [7]. Moreover, under strict infection control, unnecessary personnel such as clinical psychiatrists strongly refuse to enter COVID-19 patient’s ward [10, 11]. Natural disasters and epidemics pose many challenges in providing health care [12]. As a result, unique and innovative solutions are needed to address both the critical needs of patients with COVID-19 and other people who need healthcare service. In this respect, technological advances provide new options [13]. Although the ultimate solution for COVID-19 will be multifaceted, it is one of the effective ways to use existing technologies to facilitate optimal service delivery while minimizing the hazard of direct person-to-person exposure [7, 14]. The use of telemedicine at the time of epidemic conditions (COVID-19 pandemic) has the potential to improve research of epidemiological, control of disease and management of clinical case [7, 14, 15].

The use of telehealth technology is a twenty-first century approach that is both patient-centered and protects patients, physicians, as well as others [16, 17]. Telehealth is the delivery of health care services by health care professionals, where distance is a critical factor, through using information and communication technologies (ICT) for the exchange of valid and correct information [18]. Telehealth services are renderdusing real-time or store-and-forward techniques [19]. With the rapid evolution and downsizing of portable electronics, most families have at least one device of digital, such as smartphones [20] and webcams that provide communication between patient and healthcare provider [21]. Video conferencing and similar television systems are also used to provide health care programs for people who are hospitalized or in quarantine to reduce the risk of exposure to others and employees [7]. Physicians who are in quarantine can employ these services to take care of their patients remotely [8, 22]. In addition, covering multiple sites with a tele-physician can address some of the challenges of the workforce [8, 23].

There are various benefits in using technology of telehealth, especially in non-emergency / routine care and in cases where services do not require direct patient-provider interaction, such as providing psychological services [24]. Remote care reduces the use of resources in health centers, improves access to care, while minimizing the risk of direct transmission of the infectious agent from person to person [25]. In addition to being beneficial in keeping people safe, including the general public, patients and health workers, another important advantage is providing widely access to care givers [12].. Therefore, this technology is an attractive, effectual and affordable option [14, 26, 27]. Patients are eager to use telehealth, but hindrances still exist [28, 29]. The barriers of implementing these programs also largely depend on accreditation, payments systems, and insurance [8]. Furthermore, some physicians are concerned about technical and clinical quality, safety, privacy, and accountability [23, 30].

Telehealth can become a basic need for the general population, health care providers, and patients with COVID-19, especially when people are in quarantine, enabling patients in real time through contact with health care provider for advice on their health problems. Thus, the aim of this review was to identify and systematically review the role of telehealth services in preventing, diagnosing, treating, and controlling diseases during COVID-19 outbreak.

## Methods

### Study design

This systematic review was conducted based on the preferred reporting items for systematic reviews and meta-analyses (PRISMA) guidelines. A method of systematic review was selected to permit a robust and reproducible approach to structure a critical synthesis of the existing and current evidence. Considering the necessity of the matter and limited available evidence on the topic, we did not register the protocol of this systematic review.

### Search strategy and data sources

Five online databases, included PubMed, Scopus, Embase, Web of Science and Science Direct, were searched to identify relevant and published studies. The search was conducted on Titles and Abstracts. An elementary search in March 26, 2020 identified a range of available evidence on the role of telehealth services during 2019 novel coronavirus (COVID-19) outbreak. A further search was conducted on April 3, 2020 to update the results. The combination of keywords and Medical Subject Headings (MeSH) were used: COVID19, COVID-19, Coronavirus, Novel coronavirus, 2019-nCoV, Wuhan coronavirus, SARS-CoV-2, SARS2, Tele*, Telemedicine, Tele-medicine, Telehealth, Tele-health, Telecare, Mobile Health, mHealth, Electronic health, and ehealth. To combine terms the Boolean operators (AND, OR and NOT) were used. During this phase, a librarian was consulted to certify that the strategy of search was satisfactory. The search in each database was adapted accordingly. For example, the search strategy in the PubMed database was enforced as follows:

(COVID-19[title/abstract] OR COVID19[title/abstract] OR Coronavirus [title/abstract] OR Novel coronavirus [title/abstract] OR 2019-nCoV [title/abstract] OR Wuhan coronavirus [title/abstract] OR SARS-CoV-2[title/abstract] OR SARS2[title/abstract]) AND (Telemedicine [title/abstract] OR Tele-medicine [title/abstract] OR Telehealth [title/abstract] OR Tele-health [title/abstract] OR Telecare [title/abstract] OR Mobile health [title/abstract] OR mHealth [title/abstract] OR Electronic health [title/abstract] OR eHealth [title/abstract]).

Manual search in web-based resources was accomplished on Google, Google Scholar, journals which published key articles and through searching specific website (WHO, https://www.who.int, Centers for Disease Control and Prevention, https://www.cdc.gov, National Institute for Health and Clinical Excellence, https://www.nice.org.uk, National Health Commission of the People’s Republic of China http://www.nhc.gov and National Administration of Traditional Chinese Medicine http://www.satcm.gov.cn). In addition, we reviewed the selected articles references in order to identify additional studies or reports not retrieved by the preliminary searches (reference by reference).

### Eligibility criteria

All studies with evidence reporting the role of telehealth services in COVID-19 were included in our analyses. In fact, studies were included if they obviously defined function type of telehealth in prevention, diagnosis, management and treatment of COVID-19, published from December 31, 2019 to April 3, 2020, were written in language of English and published in peer reviewed journals. The reason for electing December 31 was due to the fact that this date coincides with the appearance of COVID-19 in Wuhan, Hubei Province, China. Actually, all studies representing any sorts of using tools of telehealth in all aspects of health care (primary, secondary or tertiary level health care) to provide clinical services, diagnosis, assessment of symptoms, triage of patients, consultation services, and training or supervision of clinicians were included. Studies about other technologies (e.g. Internet of Medical Things or IoMT), duplicate publications, review articles, opinion articles, and letters not rendering principal data were excluded, as well as studies reporting incomplete information.

### Study selection and data extraction

Two authors (A.H. and E.M.) who performed the literature search also independently followed the application of the inclusion and exclusion criteria and screened the studies based on the titles and abstracts. After initial screening, full-text of studies were obtained and examined to ensure eligibility for the development of the data extraction table.

Data were extracted from all papers which met the eligibility and inclusion criteria for the review. The following data were extracted and analyzed: first author, date of publication, country, design of study, type of used telehealth, key outputs of studies and effects of telehealth.

### Quality assessment

To assess the quality of the included studies, the Critical Appraisal Skills Program (CASP) checklists were accorded. To teach people how to critically appraise different types of evidence, the CASP tools were developed [31]. Included studies were divided into three categories of poor, medium, and good for scoring the quality of them.

### Evidence synthesis

For expressing and synthesizing the results of the included studies, narrative synthesis of overall evidence was undertaken by comparing and contrasting the data. Three stages of the narrative synthesis included the development of a preliminary synthesis, exploration of the relationships within and between studies and the determination of the robustness of the synthesis [32]. Data of the included studies was qualitatively described and presented. The authors to reach consensus on the findings, met frequently to discuss.

## Results

### Search results

**Fig. 1:**
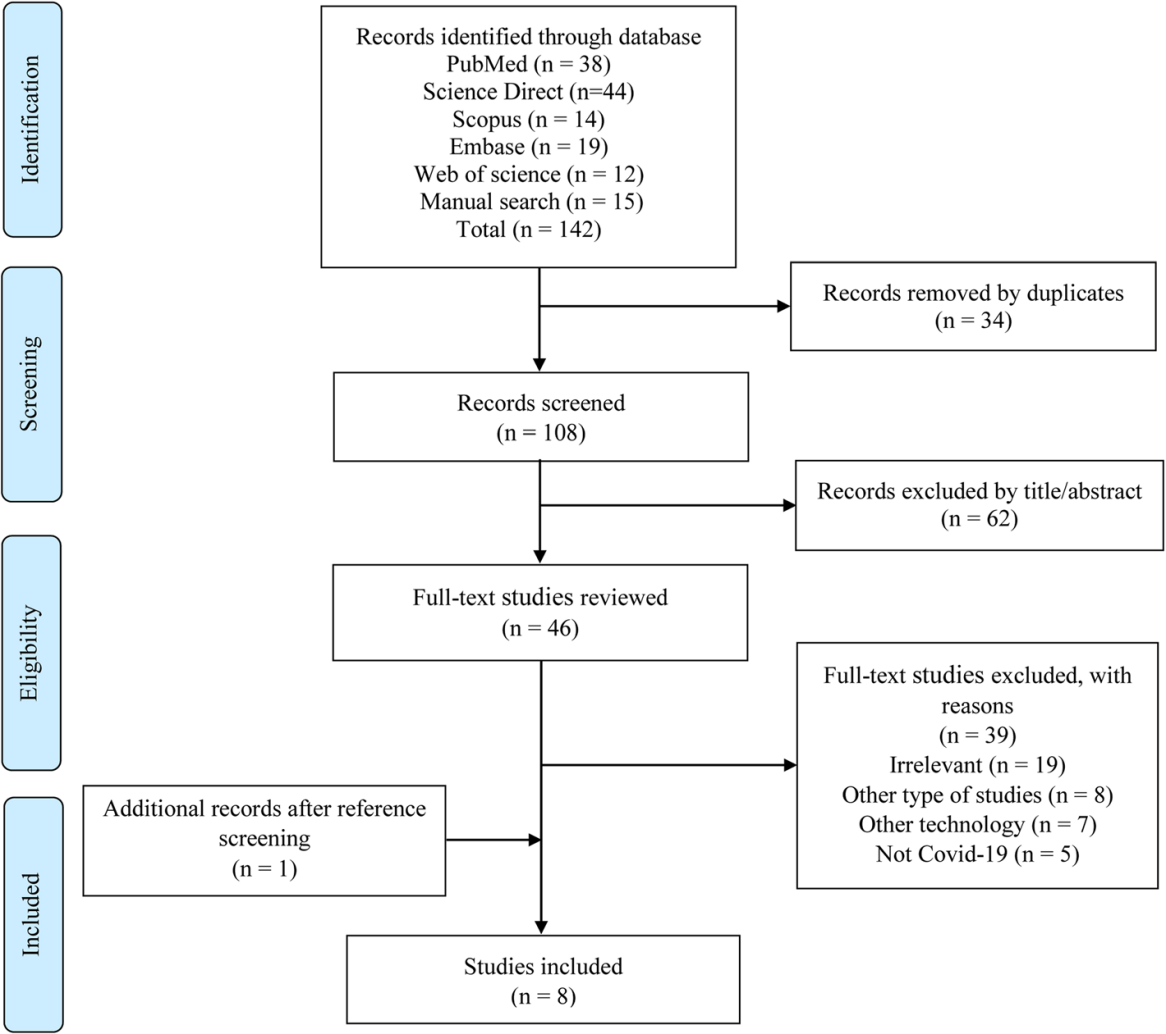
PRISMA flow diagram illustrating study selection

The details on the literature search and processes of screening are illustrate in Fig. 1. Following the removal of duplicate search records and screening titles and abstracts of studies, we appraised 46 relevant studies in full text. From the studies of remaining, 39 articles did not meet our inclusion criteria and were removed. Finally, one study was added after reference screening (reference by reference) and eight full studies included for stage of evidence synthesis.

### Characteristics of the included studies

Some general characteristics of the included studies were demonstrated in Table 1. The included studies published in various international journals between February 17, 2020 and Apr 9, 2020 were mostly conducted in the USA. Eight included studies were carried out in six countries: USA (*n* = 5), China (*n* = 2), UK (*n* = 2), Canada (*n* = 2), Iran (*n* = 1) and Italy (*n* = 1). Based on the design of study, five studies were cross-sectional, two were case studies and one was case-control. In the included studies, most of telehealth and social media channels were applied during COVID-19 pandemic such as telephone, live video conferencing, and e-mail.

**Table 1:** Summary characteristic of included studies in systematic review

Author/ Date	Country	Design of study	Type of telehealth	Key outputs	Effects
Davarpanah et al./ 17/02/2020 [33]	Iran	Case study	Social media platform including messaging software, WhatsApp, and email	• Faster rate in teleradiology services delivery • Assembled an opinion teleradiology group • Create volunteer network coordinator (humanitarian) • Triage of COVID-19 infection using radiology experts from centers from around the world	• Eliminated the need to send patients to overpopulated hospitals • Provided near real-time consultation from experts located around the country and the world • Addressed the local need • Could solve the shortage of on-site thoracic radiologists • Provided consultation in regions with limited access to thoracic radiology expertise • Established consensus among radiologists through discussions in the online group
Zhai et al./ 23/02/2020 [34]	China, UK	Case study	Live video conferencing and mobile	• Providing clinicians and patients with immediate diagnosis and consultations regarding COVID-19 • Wireless remote monitoring of patients • Remote multiple disciplinary care • Education and training of patients • Performing the collects, transforms, and evaluation of patients health data	• Led to capture, store and process patient medical records • Achieved real-time data exchange • Accessed prevention and treatment guidelines, and guidance on drug use and management of coronavirus patients • Prevented direct physical contact • Reduced the risk of exposure to respiratory secretions • Prevented the potential transmission of infection to physicians and nurses • Helped the specialist treatment team to provide primary care guidance on coronavirus for all physicians and nurses
Reeves et al./ 24/03/2020 [35]	USA	Cross-sectional	Phone calls and electronic health record (EHR)	• Triage of patient with phone calls • Screening or treating a patient in an ambulatory care setting • Screening or treating a patient in an urgent care setting • Offering decision support for those in need of testing • Repurposing and utilizing EHR optimization team to train end users’ video visit workflow • Telemedicine–video visits for outpatient clinic encounters	• Managing patients’ concerns • Tracking of COVID-related infection in EHR embedded database • Tracking of persons under investigation (PUI) in EHR embedded database • Reports regarding prior PUI, existing and pending tests, training completion and screening/documentation compliance • Updated travel and symptom screening, testing criteria, and clear guidance on best setting and location of patient care • Clinical decision support on testing criteria, recommended additional work-up, admission criteria/protocol, and discharge information • Standard documentation of any screening of patient visitors for symptoms of infection • Template excuse letter for providers to recommend working from home
Nicol et al./ 24/03/2020 [36]	USA, Canada	Cross-sectional	Social media or other digital platforms including telephone, email and videoconferencing	• Facilitating electronic informed consent, digital assessment tools and virtual study visits • E-consent, remote assessment, and telephone or videoconference visits • Provide e-consultation or advice to health providers	• Helped in implementing social distancing • Could be implemented far from high-risk areas such as hospital grounds • Reduced the use of public transportation • Provided all of the components of human research protection • Reduced viral transmission risk from in-person contacts • Prevented morbidity in these at-risk individuals during the COVID-19 pandemic • Communicated accurate and clear information, at a time when older adults and their family are bombarded with contradictory and confusing messages
Simcock et al./ 24/03/2020 [37]	UK, USA, Italy	Cross-sectional	Telephone, video, and laptops	• Telephone follow up in multiple cancer settings (endometrial, prostate, lung, and colorectal cancer) • Use in remote monitoring • Provide video consultations	• Minimized the risk of COVID-19 transmission during radiotherapy treatment • Reduced infection risk and the risks of workforce depletion • Facilitated access to hospital data or to treatment planning systems
Greenhawt/ 26/03/2020 [38]	USA, Canada	Cross-sectional	Telephone, electronic medical record, patient portal messaging, digital photography, video using a HIPAA-compliant platform, website	• Delivering allergy services • Phone triage as available options in allergic rhinitis • Provide telehealth visits • Follow-up visits, via phone triage or telehealth in patients with urticaria, angioedema, and atopic dermatitis • Service adjustment for food allergy, Eosinophilic Esophagitis (EoE), drug allergy, and anaphylaxis • Service adjustment for allergic skin disorders and • Service adjustment for immunodeficiency • Immunotherapy appointments or schedules • Served as a portal for sharing timely information to large numbers of patients	• Limited the exposure of providers to potentially infected patients • Provided access to rapid evaluation for potential COVID-19 infection • Reduced exposure of patients • Preserved social distancing • Could meet healthcare needs • Helped visualize any rash • Reduced the need for face-to-face visits • Virtual care options to ensure continuity of care • Was Effective for managing patients with chronic conditions • Provided an opportunity to introduce telehealth in to an allergy practice • Reduced burden on practice resources
Cohen et al./ 07/04/2020 [39]	USA	Cross-sectional	Applications including Apple FaceTime, Facebook Messenger video chat, Skype, and Mobile health technology	• To develop staffing plans • Using to conducted billing of patients • To appropriately-performed telehealth visits • Use in psychological treatments • contact with family, friends and colleagues • In-person evaluation, telemedicine evaluation if high-risk for infection (patient or location-specific)	• Minimized “unnecessary” exposure of hospital staff to patients, and to themselves • Led to early treatment associated with better outcomes
Zhou et al./ 09/04/2020 [40]	China	Case-control	combined mode of MOOC micro-video	• The live broadcast of the training video • Can be watched repeatedly videos • Was applied to the communication ability training of new nurses	• Satisfaction was higher • Understanding was easy • The teachers’ evaluation and harvest were higher • Obtained the real clinical experience • Helped to alleviate the lack of clinical nursing teaching resources

### Quality assessment

Our systematic review included eight studies that were appraised using the tools of CASP. The qualities of the assessed studies were generally in high level. Six (75%) studies enjoyed good quality, and two (25%) had medium quality. Also, no studies were excluded on the basis of the level of quality appraisal.

### Telehealth services during the COVID-19 outbreak

We recognized eight studies that presented precious data on telehealth regarding the status of people infected with COVID-19. Telehealth has the capability to incorporate several organizations and situations of health care into one virtual network, led by the central clinic. This network can contain physical locations in different region: central and remote clinics, prevention centers, private clinics, and, private offices of physicians, centers of rehab state and all registered patients within their locations. By using virtual care for very regular, essential medical care, and deferring elective procedures or yearly checkups, we can free up medical staff and equipment required for those who become seriously ill from COVID-19. Additionally, by not congregating in small spaces like waiting rooms, the ability of the coronavirus to transmission from one person to another were thwart. Keeping people discrete is called “social distancing”. Keeping healthcare staffs discrete from patients and other providers is “medical distancing”. In present time the Telehealth is one strategy to help us carry out this.

Telehealth can mobilize all aspects of healthcare potentials to decrease transmission of disease, conduct people to the right level of health care, ensure safety for provide health services online, protect patients, clinicians, and the community from exposure to infection, and finally diminish the burden on the healthcare providers and health system. Some of the telehealth usage cases for patients were control and triage during the outbreak of COVID-19 pandemic, self and distance monitoring, treatment, patients after discharge in health centers (follow-ups) and implementation of online health services. These methods have the potential to reduce morbidity and mortality during pandemic. For all healthcare workers and clinicians with mild symptoms can still work remotely with patients, facilitate quick access to medical decision making, seek second opinion for severe cases of patients, exchange cross-border experiences, and offer teleradiology and online trainings for health workers. To provide continued access to necessary health services, telehealth should be a key weapon in the fight against the COVID-19outbreak.

## Discussion

The aim of this systematic review was to identify the role of telehealth services in preventing, diagnosing, treating, and controlling diseases during COVID-19 pandemic. In this review, we explained the benefits and implications of several tools of telehealth with the purpose of improving the management of COVID-19 infection. Nowadays, the best preventive strategy is to avoid being exposed to the coronavirus, because there is no vaccine to overcome COVID-19 in the all countries [41]. A series of strategies have been proposed for infection prevention and control (IPC) that may diminish the exposure risk, such as wearing of face masks in mass population, Covering mouth and nose with tissue when coughing and sneezing, continuous hand washing with soap and water or hand sanitizer containing at least 60% alcohol, avoidance of close contact with others and keeping true social distance, and refraining from touch of unwashed hands with eyes, nose, and mouth [42].

Meanwhile, to reduce the number of those who receive face-to-face services of health care, healthcare workers can contact with patients through telecommunication tools for triaging, assessing and caring for all patients [43]. Telehealth with use of live video conferencing or a simple mobile call allow health care professionals to ask special questions and collect required information, triage of patient and supply consultation, or if a person can continue to self-monitor symptoms at home while recovering. It can also be applied for regular check-ins such as respiratory, blood pressure and oxygen level rate needed in home [34].

During the COVID-19 outbreak in China, online mental health surveys with communication programs, such as Weibo, WeChat and TikTok have enabled mental health professionals and health authorities to render safety mental health services online during the COVID-19 pandemic [44]. Chinese government officials launched a remote consultation network that can be carried out internet or telephone consultations in a safe setting to ensure the continuous provision of mental health services and reduce the hazard of cross infections [45]. Also, the National Health Commission of China have published several online guideline and free electronic books about COVID-19 with the aim of helping the progress of Chinese people emergency interventions, safety, improving the quality and effectiveness of emergency interventions [10]. In addition, telehealth can provide mental online health services in the setting of patient isolation by reducing the mental health burden from COVID-19 and sharing information about symptoms of burnout, depression, and anxiety [14].

Greenhawt et al. suggested that telehealth has several benefits in providing allergy and immunology services such as limiting exposure of health professionals to potentially infected patients and access to the rapid evaluation for COVID-19 infection [38]. in addition to the conventional methods used in diagnosing COVID-19, the study identified a novel screening and triage strategy during deadly COVID-19 pandemic in Iran. Services for teleradiology and teleconsultation for triage of COVID-19 infection through a social media massager delivered by Iranian Society of Radiology (ISR) to response to the shortage of on-site thoracic radiologists during COVID-19 pandemic [33]. In addition to taking actions to protect the health and safety of patients, also staff should take mobile health technology to develop staffing plans and carry out billing for healthcare services [39].

Our results demonstrate that to manage COVID-19, there are many easy-to- set-up potentials in live video consulting. Live video conferencing can lead to the avoiding of direct physical contact, thereby diminishing the risk of exposure to respiratory secretions and preventing the potential transmission of infection to physicians and other healthcare providers [34]. Also, live video could be very useful for patients seeking consultation on covid-19, for people with heightened anxiety and instead of in-person visits in cases of chronic disease reviews (such as diabetes and cancer), some medication checks, and triage when telephone is insufficient [23]. In order to control the spread of the COVID-19 outbreak, video consultations and telephone follow-up is possible in multiple cancer settings including lung, endometrial, colorectal, and prostate [37].

Based on the study conducted in the USA, phone calls and electronic health records (EHR) can facilitates screening or treating a patient without the need for in-person visits and improve decision making process among healthcare team in an ambulatory and urgent care [35]. Generally, the impact of telehealth during the COVID-19 pandemic in preventing morbidity and avoiding of presence the public from high-risk areas such as hospital premises was significant. Also, the elderly people can access health services by using electronic devices [36]. These days, suitable adaptation of local systems with changes regarding to payment and coordination of services are major barriers for the large-scale use of telehealth to deal with COVID-19 infection [8]. Finally, we hope can make substantial progress in preventing and controlling COVID-19 pandemic through further training of health providers and patients on how to make the most of telehealth tools, revisiting traditional definitions of clinical practice and using closed online platforms.

### Future research

The biggest challenge for future research in the use of telehealth is probably defining the obstacles and facilitators in health providers and patients. Future research is suggested to specify the effects of telehealth solutions in the efficiency indicators and hospital performance. Also, further global research is warranted to determine how to set up telehealth in primary care. Researchers can also examine the effectiveness of using telehealth approaches in different health areas, especially in the field of home nursing the elderly who are high-risk people in the community. It is also highly recommended to use this technology in the field of psychiatry as it does not require in-person visits. Other future research can tap into evaluating the satisfaction of patients and providers with telehealth services.

### Limitations

Our systematic review holds three limitations. Firstly, it is possible that some relevant studies were not taken into account because they have been published in languages other than English (e.g. Chinese). Secondly, we did not have access to some other databases such as CINAHL and PsycINFO. Thirdly, there could be some other studies on this theme in the literature that skipped our attention and analyses though we did our best to adopt a comprehensive search strategy and cover a broad range of evidence across the world.

## Conclusion

This study provides a comprehensive systematic review solely exploring the potentials of telehealth during the COVID-19 pandemic. In response to WHO’s call for studies on the COVID-19 infection and presentation of the most recent evidence published in this early period of the outbreak for health care providers, this study was conducted to identify the role of telehealth during COVID-19 outbreak. As the COVID-19 epidemic scales exponentially across the worlds, calls for expended use of telehealth as innovative solutions, clearly highlight unmet needs in the world healthcare system. Telehealth has the potential to address many of the key challenges in providing health services during the outbreak of COVID-19. Also, telehealth can help us avoid direct physical contact and minimize the risk of COVID transmission and finally provide continuous care to the community. Based on the findings of this review study, clinicians and patients are strongly recommended to apply telehealth tools as an appropriate option to prevent and contain COVID-19 infection.

## Data Availability

Datasets are available through the corresponding author upon request.

## Abbreviations

COVID-19: Corona virus disease 2019
CASP: Critical appraisal skills program
WHO: World health organization
MeSH: Medical subject headings
IoMT: Internet of medical things
IPC: Infection preventive and control
HER: Electronic health record
PUI: Persons under investigation
